# Identification and validation of obesity related genes signature based on microenvironment phenotypes in prostate adenocarcinoma

**DOI:** 10.18632/aging.205065

**Published:** 2023-10-02

**Authors:** Linghui Liang, Jinwei Shang, Yuwei Zhang, Yuxin Xu, Yihui Zhouteng, Jianxiang Wen, Yuxin Zhao, Ninghan Feng, Ruizhe Zhao

**Affiliations:** 1 Department of Urology, Affiliated Wuxi No. 2 Hospital, Nanjing Medical University and Jiangnan University Medical Center, Wuxi, Jiangsu, China; 2 Department of Urology, The First Affiliated Hospital of Nanjing Medical University, Nanjing, Jiangsu, China; 3 Nantong University Medical School, Nantong, Jiangsu, China; 4 Nanjing Medical University, Nanjing, Jiangsu, China

**Keywords:** obesity, prostate adenocarcinoma, immunotherapy, prognosis, tumor microenvironment

## Abstract

Background: The role of obesity related genes (ORGs) in the immune checkpoint inhibitors (ICIs) treatment of prostate adenocarcinoma (PRAD) has not yet been proved by research.

Methods: We comprehensively evaluated the ORGs patterns in PRAD based on tumor microenvironment (TME) phenotypes and immunotherapy efficacies. Then we constructed a ORGs risk score for prognosis and a ORGs signature for accurate prediction of TME phenotype and immunotherapy efficacy in order to evaluate individual patients.

Results: Two distinct ORGs patterns were generated. The two ORGs patterns were consistent with inflammatory and non-inflammatory TME phenotypes. ORGs patterns had an important role for predicting immunotherapy efficacies. Next, we constructed a ORGs risk score for predicting each patient’s prognosis with high performance in TCGA-PRAD. The ORGs risk score could be well verified in the external cohorts including GSE70769 and GSE21034. Then, we developed a ORGs signature and found it was significantly positively correlated with tumor-infiltrating lymphocytes in TCGA-PRAD. We found that each patient in the high-risk ORGs signature group represented a non-inflamed TME phenotype on the single cell level. The patients with high ORGs signature had more sensitivity to immunotherapy. And those ORGs were verified.

Conclusions: ORGs pattern depicts different TME phenotypes in PRAD. The ORGs risk score and ORGs signature have an important role for predicting prognosis and immunotherapy efficacies.

## INTRODUCTION

Prostate cancer (PCa) is the second most common malignancy and the sixth leading cause of cancer deaths in the world [1]. Due to the growth and aging of the population, the global burden of PCa is increasing year by year. Some epidemiological studies have shown that obesity is associated with increased risk and death of many types of cancer, including PCa in recent years [2, 3]. In addition, dysfunctional adipose tissue behavior, often seen in obesity, has been widely recognized as the main cause of cancer [4]. Obesity affects many men just like PCa. Two-thirds of Americans are classified as overweight and one-third as obese. These trends have stabilized and become a permanent feature of American society [5]. Similarly, in Europe, the prevalence of overweight and obesity continues to rise [6].

Since both obesity and PCa affect a considerable number of men, the association between them is of great significance. Although in the era of PSA, obesity may be negatively related to the overall risk of PCa, it has a certain correlation with the increased risk of death of PCa, the pathological characteristics of poor prognosis and the risk of biochemical recurrence after radical prostatectomy [7].

Anatomically, the prostate is a capsule-like structure surrounded by adipose tissue. Physiologically, prostate tumor cells often infiltrate the periprostate fat pad through transposition or infiltration capsule [8], which lead to invade adipose tissue directly. If PCa cells extend beyond the capsule, factors secreted by periprostate adipose tissue around the prostate, extracellular matrix components or direct cell-cell contact may affect the phenotypic behavior of malignant cells.

More and more attention has been turned to elucidate the potential molecular mechanism. The study in periprostate adipose tissue found that tumor related factors affect its metabolic activity and increase the production of local adipose factors and the thickness of periprostate fat, which are related to the invasiveness of PCa [9–12]. Adiponectin and leptin are proteins synthesized by adipocytes and involved in energy regulation and apoptosis [13]. There are even some research evidences that there is a correlation between adipocyte-derived proteins and the risk or severity of PCa [14]. Obesity is a risk factor for invasive PCa [2, 15]. PPARG affects insulin sensitivity, while TCF7L2 affects insulin secretion. Because of the negative correlation between type 2 diabetes and PCa, both may affect the risk of PCa [16]. The variants of these genes have been confirmed and are related to diabetes [17, 18].

Tumor microenvironment (TME) is composed of malignant cells and non-malignant cells. Based on the existence of tumor infiltrating lymphocytes (TILs), it can be divided into two phenotypes. Inflammatory tumors are tumors with high TILs infiltration, while non-inflammatory tumors are tumors with low infiltration [19]. For tumors with a high degree of TILs invasion, patients will have a significantly higher response to immune checkpoint inhibitors (ICIs) treatment [20, 21]. At present, mCRPC clinical trials use a mixture of ICIs as an alternative method [22]. The increase of body mass index caused by obesity leads to various metabolic disorders, which affect the TME and then promote the occurrence of tumors [23]. Therefore, we hypothesized that obesity related genes (ORGs) also affect TME. However, the comprehensive analysis of these ORGs in prostate adenocarcinoma (PRAD) is still lacking.

In this study, we firstly used comprehensive bioinformatics analysis to correlate ORGs with TME phenotype, precise immunotherapy efficacy and prognosis in PRAD.

## MATERIALS AND METHODS

### Data sources

From UCSC Xena (https://xenabrowser.net/) downloaded TCGA-PRAD’s the fragments per kilobase per million mapped fragments (FPKM), count value and clinical data. We transformed FPKM value into transcript of one thousand base millions (TPM) value, and exclude duplicate patients or patients without matching RNA seq data and survival data. Finally, 494 patients in TCGA-PRAD were used for further analysis. And GSE70769 and GSE21034 were downloaded from GEO database (https://www.ncbi.nlm.nih.gov/geo/).

### Unsupervised clustering

The obesity related genes (ORGs) included genes associated with monogenic obesity and the leptin-melanocortin signalling pathway, genes associated with insulin signalling pathway, genes associated with lipid metabolism, other genes associated with appetite regulation. They were collected from the studies of Catarina et al. [24] and Ricardo et al. [25]. We used the “ConsensuClusterPlus” R package (maxK=4, reps=1000, pItem=0.8, distance=“euclidean”, clusterAlg=“km”) to perform consensus clustering and repeat 1000 times. ORGs were summarized in Supplementary Table 1.

### Pathway enrichment analysis

We collected 4 immune-related signatures and 21 signatures related to the efficacy of immune checkpoint blockade (ICB) therapy from previous studies [26–28]. Then, we used the single sample gene set enrichment analysis (ssGSEA) implemented in the “GSVA” R package to calculate the sample level enrichment scores of these signatures. The differential gene expression analysis adopted empirical Bayesian algorithm (“limma” R package). The standard of differential expression genes (DEGs) was set as absolute log2 fold change (FC) greater than 2.5, and the adjusted *p*-value was less than 0.05. From MSigDB [29] (http://www.gsea-msigdb.org/gsea/index.jsp) downloaded the Hellmark, gene ontology (GO) and Kyoto Encyclopedia of Genes and Genomes (KEGG) gene sets, and then conducted GSEA analysis using the “GSVA” R package [30].

### Tumor immune microenvironment depiction

The anti-cancer immunity cycle was a seven step anti-tumor immune cell activation process, so we downloaded the levels of each step from the tracking tumor immunophenotype (TIP) (http://biocc.hrbmu.edu.cn/TIP/) [31]. Based on the gene set reported by Sharoneton, we used the ssGSEA algorithm to calculate the relative abundance of 28 immune cells in TCGA-PRAD and GEO cohorts [32].

### Construction and verification of ORGs model

The TCGA-PRAD, GSE70769 and GSE21034 datasets were divided into training and verification sets according to the inclusion of patients in the experiment. In the training set (TCGA-PRAD), we used univariate Cox analysis to analyze common DERs, and then used LASSO algorithm to screen the best candidate DERs. Next, we used multivariate Cox regression coefficient to select the best candidate DERs, and established ORGs model based on ORG RNA expression pattern and weighted using the formula: ORGs=Σβi * RNAi, where βi represented the expression pattern coefficient of the ‘i’th ORG RNA. Finally, logarithmic rank test and Kaplan-Meier analysis were employed to evaluate the prognostic predictive value of ORGs model.

### Single cell RNA sequencing

We downloaded the scRNA-seq dataset containing two PRAD samples from the Supplementary Materials of GSE157703 [33]. Then we created a Seurat object by using the “Seurat” R package on the raw count matrixes, and set the inclusion criteria for high-quality cells as follows: the numbers of unique molecular identifiers (UMI) more than 1000, the number of genes more than 250, the log10GenePerUMI more than 0.80, and the percentage of mitochondria less than 20%. Next, the raw data count was normalized, identified variable genes, and scaled by using the SCTransform function. Based on the Anchors generated by top 3000 variables (FindIntegrationAnchors function), 2 samples were integrated. After integration, we used the RunPCA function for principal component analysis (PCA), and then used the top 40 PCs to perform manifold approximation and projection (UMAP) reduction. At the same time, we used the FindClusters function to identify the main cell clusters with a resolution value of 0.4, and annotated the cell clusters according to the gene markers of the relevant research [34]. Finally, we used the AddModoleScore function to generate ORGs signature at the single cell level. In addition, based on GSE70769 and GSE21034, the effectiveness of ORG model in predicting prognosis was verified.

### Cell culture

Human normal prostate cell lines (RWPE-1) and PCa cell lines (22RV1 and PC3) were from China Center for Type Culture Collection and cultured in RPMI 1640 (Gibco, Grand Island, NY, USA), which contained 10% fetal bovine serum (Gibco, Grand Island, NY, USA), 100U/ml Penicillin and 100mg/ml streptomycin (Invitrogen). They both were cultured in a humidified incubator at 37° C and 5%CO_2_.

### RNA isolation, cDNA synthesis, and RT-qPCR

Total RNA was isolated and extracted by TRIzol reagent (Thermo, Waltham, MA, USA). Then we used the ReverTra Ace qPCR RT kit (Toyobo, Osaka, Japan) to synthesize the first-strand cDNA from 1 μ g total RNA. Fast SYBR Green Master Mix was used for RT-qPCR (Applied Biosystems, Foster City, CA, USA). The cycle conditions were that the polymerase was activated at 95° C for 30 s followed by 40 cycles at 95° C for 5s and 60° C for 30s. And we use GAPDH as an internal loading control. The relative level was calculated by the relative quantification 2-∆∆ CT method. All the primers were as listed as (Supplementary Table 2).

### Statistical analysis

The unpaired t-test was used to compare the differences of continuous variables with normal distribution, and the Wilcoxon rank-sum test was used to compare the differences of continuous variables with non-normal distribution. χ2 or Fisher’s exact test was used to compare categorical variables. Then we used Kaplan-Meier method and log-rank test (“surveyor” R package) to generate survival curves and survival differences of the two groups of patients. Pearson correlation coefficients were used for correlation analysis. Time dependent receiver operating characteristic (ROC) analysis (timeROC function in “tROC” R package) was used to judge the accuracy of prediction. Furthermore, we adjusted the *p*-value for DEG and GSEA analysis using the false discovery rate (FDR) method. The criteria of the significant difference were that the two-tailed *p*-value was less than 0.05. All analyses were performed using R 4.1.2.

### Data availability statement

The data of this study were from publicly available datasets. They could be found here: https://portal.gdc.cancer.gov/; https://www.ncbi.nlm.nih.gov/geo/.

## RESULTS

### Obesity related genes (ORGs) regulated patterns in PRAD

**Figure 1:**
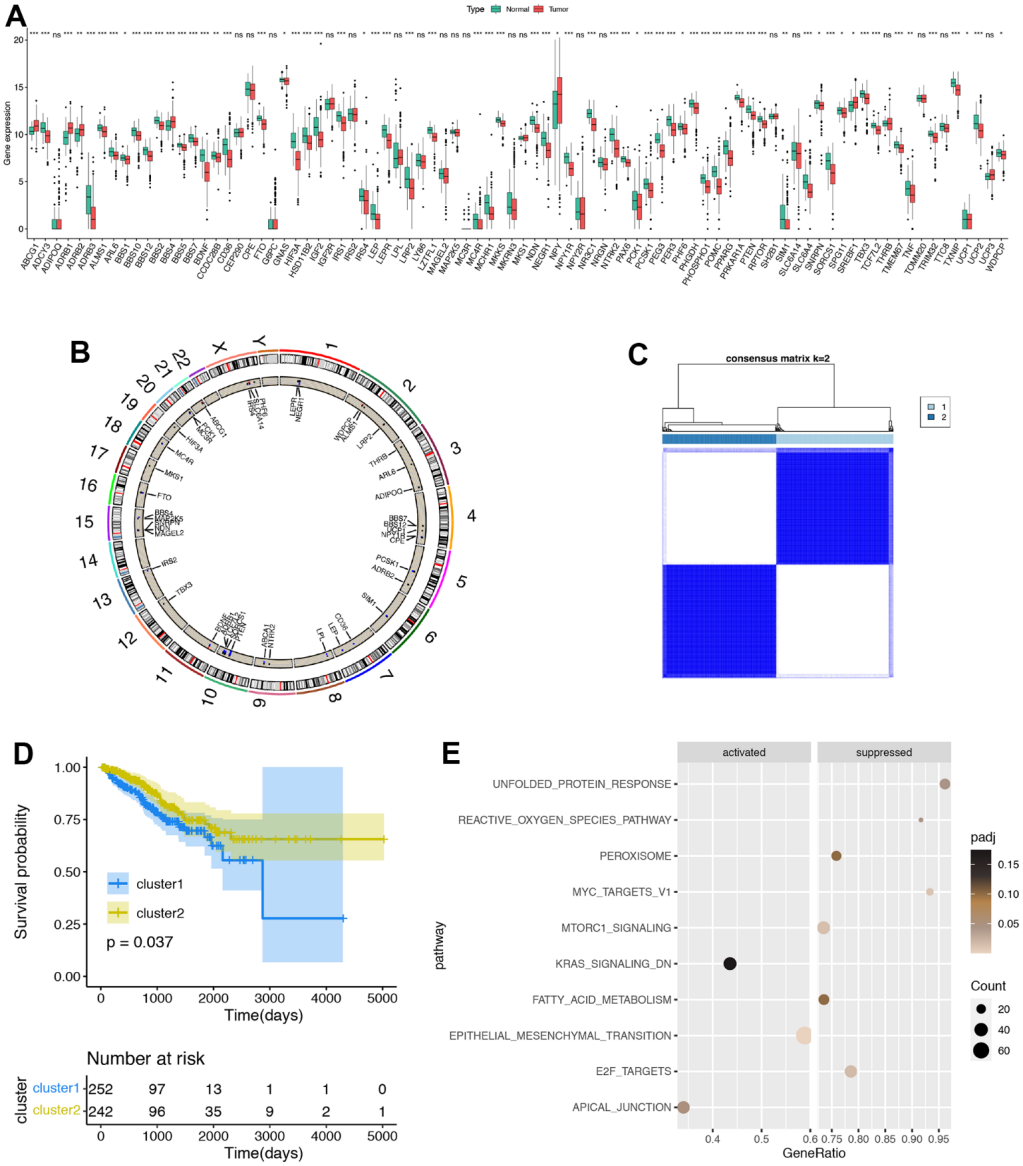
**Development of obesity related genes (ORGs) regulated patterns in prostate adenocarcinoma (PRAD).** (**A**) The expression of ORGs in PRAD and normal prostate tissues, Normal, blue; Tumor, red. (**B**) The comprehensive interactions between ORGs. The size of circles represented the different effects of genes on the prognosis. Blue dots in the circles showed favorable factors for progression free survival (PFS), while red dots showed risk factors. (**C**) Two clusters were generated by unsupervised clustering based on those ORGs. (**D**) Kaplan-Meier plots between two ORGs regulated patterns. Blue line showed ORGs cluster 1, while red line showed ORGs cluster 2. (**E**) Gene set enrichment analysis (GSEA) of hallmark pathways between ORGs cluster1 and cluster2. *p < 0.05; **p < 0.01; ***p < 0.001; ****p < 0.0001. ns, not significant.

ORGs had dysregulation between PRAD and normal tissues (Figure 1A). In these dysfunctional ORGs, combined with relevant researches, ABCG1 is a potential target for treating ovarian cancer associated with ECM1-activated signaling [35], while LEPR is the one of breast cancer risk factors [36], indicating ORGs might play important roles in PRAD. Furthermore, we described the CNV alterations locations of ORGs on chromosomes (Figure 1B). Based on these results, we analyzed whether ORGs had a comprehensive regulatory pattern in PRAD and performed unsupervised clustering. In addition, TCGA-PRAD patients could be well divided into two clusters, namely ORGs cluster 1 and cluster 2 (Figure 1C). Significantly, patients in ORGs cluster 2 shown significantly favorable survival probability than patients in cluster 1(Figure 1D). The GSEA results of the landmark pathway showed that some epithelial-mesenchymal transition (TME) related pathways were activated in cluster 2 (Figure 1E), revealing that there might be different EMT states between the two clusters.

### Different immune characteristic between ORGs regulated patterns

**Figure 2:**
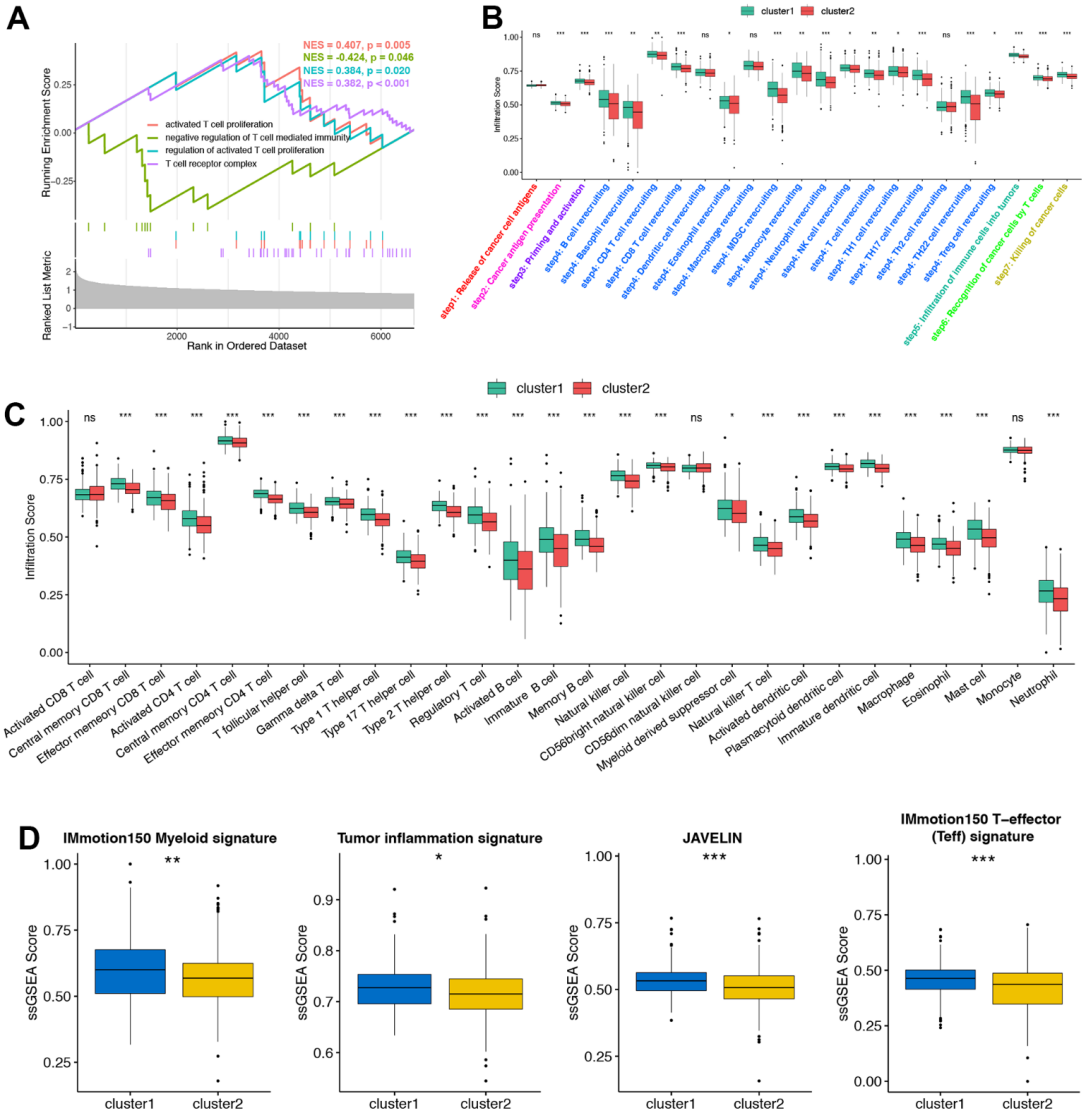
**Consistency between ORGs patterns, tumor microenvironment (TME) phenotypes and immunotherapy efficacy.** (**A**) T cell regulated pathways in gene ontology (GO) pathways using GSEA analysis. (**B**) The location of CNV alteration of ORGs on 43 chromosomes in the PRAD cohort. (**C**) Different infiltration status of immune cells into TME between two ORGs regulated patterns. Tumor, red; Normal, azure. (**D**) Box plots of IMmotion150 Myeloid signature, Tumor inflammation signature, JAVELIN and IMmotion150 T-effector (Teff) signature respectively between two ORGs regulated patterns. *p < 0.05; **p < 0.01; ***p < 0.001; ****p < 0.0001. ns, not significant.

In ORGs cluster 2, the positive regulation of most T cell activation pathways could be significantly suppressed (Figure 2A and Supplementary Table 2). In order to understand whether cluster 2 can represent the non-inflammatory TME phenotype of PRAD, we compared the cancer immune cycle between the two clusters. Figure 2B shown that most cancer immune cycles in cluster 2 are significantly lower than cluster 1, suggesting that patients in cluster 2 might inhibit cancer immune activation and immune cell infiltration into TME. According to ssGSEA results, we found that most TILs like activated CD4+T cells, dendritic cells (DCs), CD8+T cells and natural killer (NK) cells were significantly reduced in ORGs cluster 2 (Figure 2C). These above results supported that cluster 2 represented the non-inflammatory TME phenotype and was not sensitive to ICIs treatment, while cluster 1 represented the inflammatory phenotype and might be sensitive to ICIs treatment. At the same time, we found that four immune related pathways, including IMmottion150T-effector (Teff) signature, IMmottion150 Myeloid signature, JAVELIN signature and Tumor inflammation signature (TIS), were significantly suppressed in ORGs cluster 2 (Figure 2D). The above results showed that ORGs cluster 2 could not be sensitive to ICIs treatment. But unsupervised clustering was performed based on a cohort of patients, which could not evaluate the regulation pattern of a single patient. Therefore, our aim was to screen new genes to predict the prognosis of individual patients, the infiltration of TILs and the efficacy of immunotherapy.

### Construction and validation of ORGs risk score and its clinical significance

**Figure 3:**
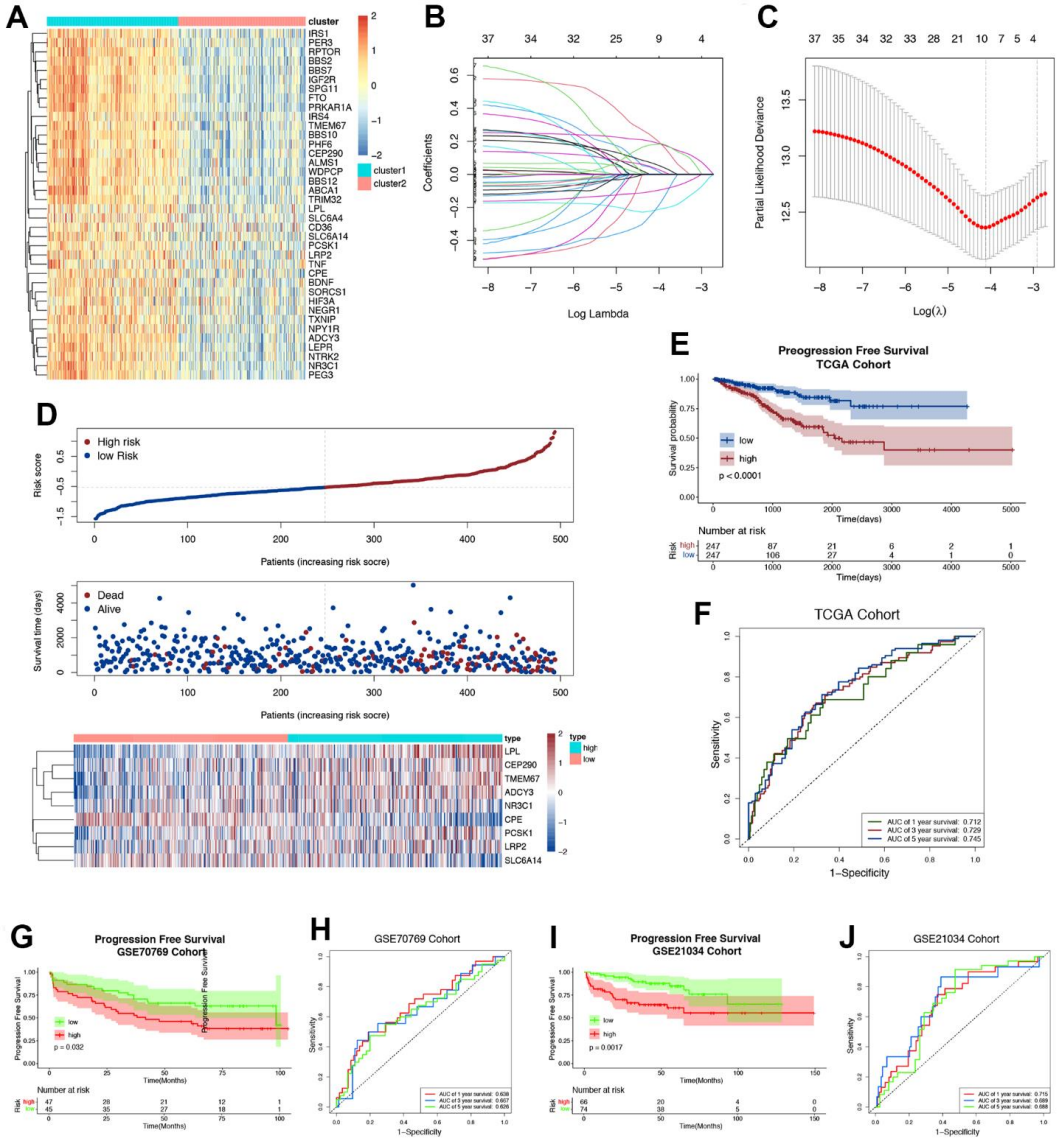
**Construction and validation of ORGs risk score.** (**A**) The expression levels of ORGs in two clusters. The darker color indicates a higher expression, where up-regulated genes were marked as red, and down-regulated genes were marked as blue. (**B**) LASSO regression of the ORGs possessing prognostic value. (**C**) Cross-validation for turning parameter selection via minimum criteria LASSO regression model. (**D**) Distribution of risk score, survival status and the expression of nine prognostic ORGs in PRAD. (**E**) Progression free survival (PFS) for PRAD patients in high-/low-risk group. (**F**) The ROC curve of measuring the predictive value. (**G**) The Kaplan-Meier survival plot of each patient in GSE70769 cohort. (**H**) The time-dependent ROC curves of ORGs risk score in GSE70769 cohort. (**I**) The Kaplan-Meier survival plot of each patient in GSE21034 cohort. (**J**) The time-dependent ROC curves of ORGs risk score in GSE21034 cohort.

The RNA levels of ORGs were presented as heatmaps in Figure 3A. These ORGs were enriched or downregulated in cluster 1 and cluster 2, respectively. The ORGs model was based on these ORGs (Figure 3B, 3C). By implementing the LASSO Cox regression analysis, the gene signature was built based on the optimum λ value. We divided 494 patients into low-risk and high-risk subgroups according to the median score calculated by the risk scoring formula. Compared with patients in the low-risk group, the patients of high-risk group had more deaths and shorter survival time (Figure 3D). In the TCGA training group, the low-risk group showed a significant progression free survival advantage compared with the high-risk group (Figure 3E). The AUC of the ORGs model in 1-, 3-, and 5-year survivals was 0.712, 0.729, and 0.745, respectively (Figure 3F). We also found that ORGs had great potential in predicting the prognosis of GSE70769 validation cohort and GSE21034 validation cohort (Figure 3G–3J).

### The signatures of ORGs could better predict PRAD prognosis in clinical scenarios

**Figure 4:**
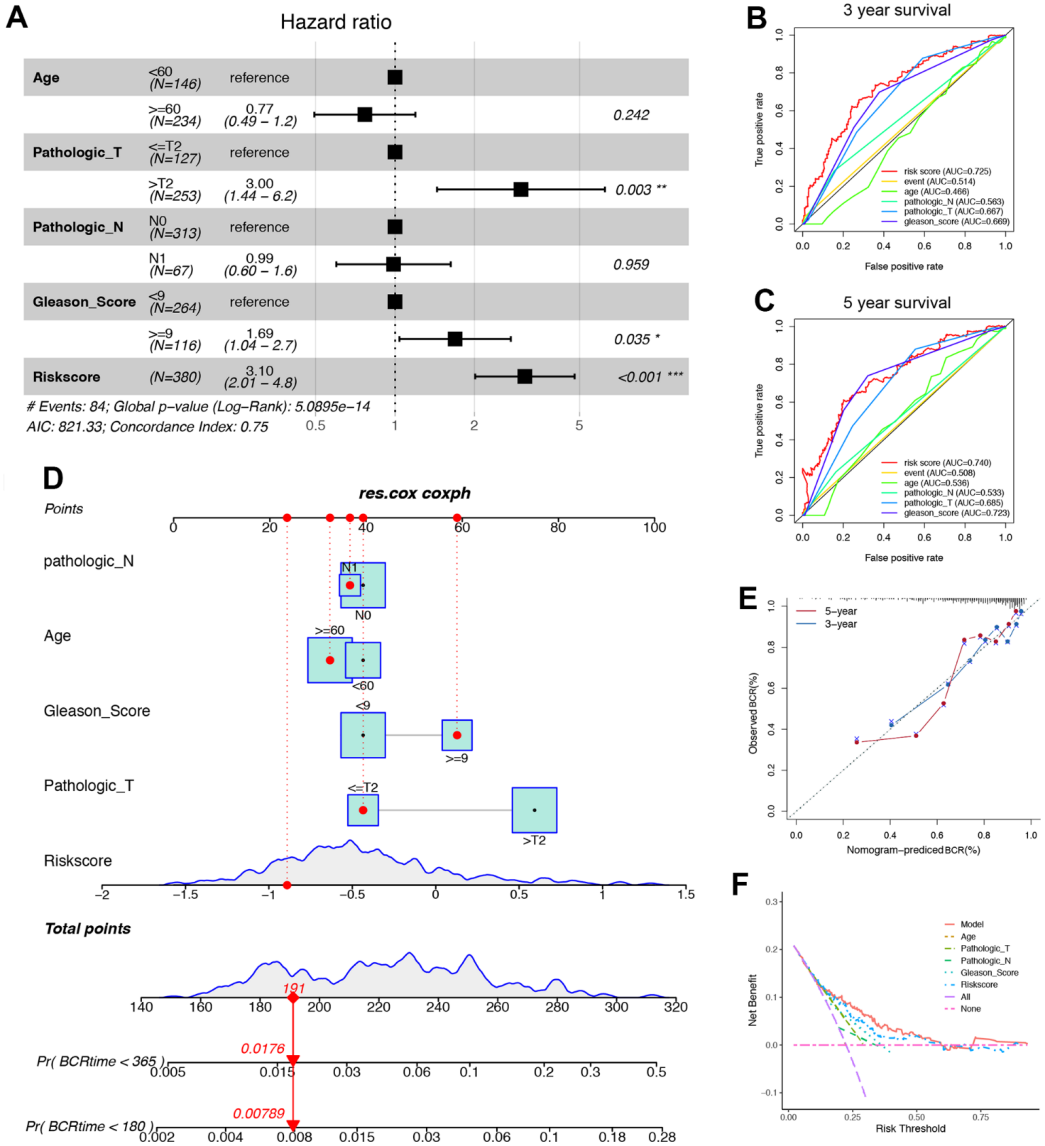
**ORGs risk score associated with immune microenvironment is a valuable prognostic model in TCGA cohort.** (**A**) In the multivariate COX regression, age, Pathologic_T, Pathologic_N, Gleason_score and ORGs risk score were used to construct a forest map. (**B**) The ROC curve of indicating that the risk score was better than other models for predicting the 3-year survival condition of PRAD patients. (**C**) The ROC curve of indicating that the risk score was better than other models for predicting the 5-year survival condition of PRAD patients. (**D**) A nomogram was constructed using independent prognostic factors such as age, Pathologic_T, Pathologic_N, Gleason_score and ORGs risk score. (**E**) The calibration diagrams were applied to evaluate 3- and 5-years overall survival probabilities. (**F**) The Net Benefit plot showed that the ORGs model had better net benefit than other models in the quantity of false positives.

We investigated whether ORGs signatures could better predict the clinic-pathological characteristic of PRAD. Firstly, the nomogram was constructed to predict 180- and 365-days biochemical recurrence (BCR) using 380 PRAD cases by incorporating prognostic factors, including age, pathological T stage, pathological N stage, Gleason score and risk score (Figure 4A, 4D). Then the ROC analysis indicated that the risk score was better than other models for predicting the 3- or 5- survival condition of PRAD patients (Figure 4B, 4C). And calibration plot was drawn to show the possibility of overestimation or underestimation of mortality by the nomogram (Figure 4E). Finally, Figure 4F showed that the ORGs model had better net benefit than other models in the quantity of false positives.

The prognostic value of ORGs risk score algorithms was evaluated. The results showed that ORGs risk score had superior performance in predicting BCR prediction to other factors for GSE70769 and GSE21034 cohorts (Supplementary Figure 1). Based on these results, the risk score of ORGs model could play a notable role in clinical prediction.

### ORGs signature for immune cell infiltration evaluation

**Figure 5:**
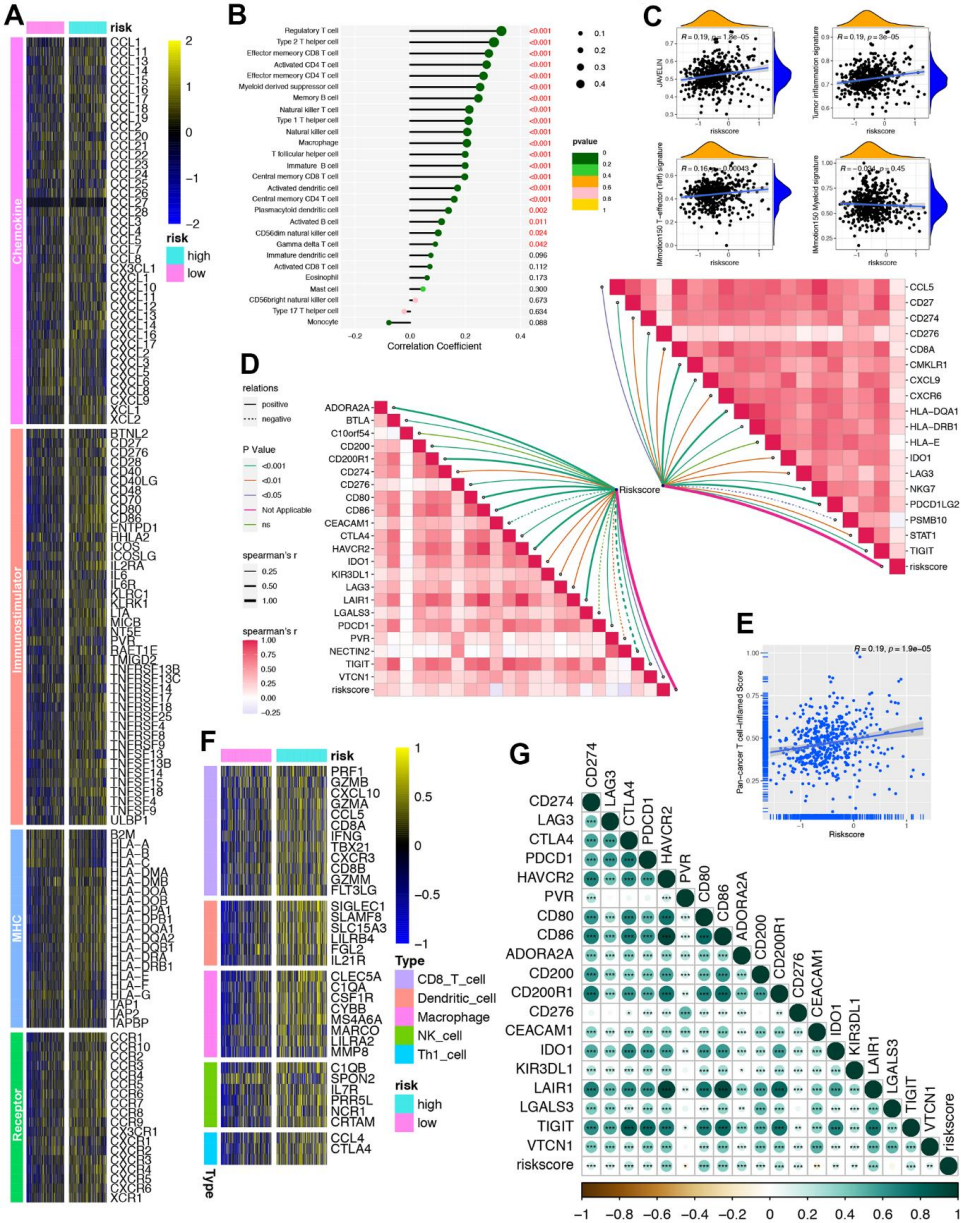
**Developing a ORGs signature for individual patient’s tumor microenvironment (TME) phenotypes evaluation.** (**A**) Differences in the expression of 122 immunomodulators (chemokine, receptor, MHC and immunostimulators) between high-risk and low-risk cohorts in PRAD. (**B**) Correlation between ORGs signature and immune cells infiltration in TCGA-PRAD. (**C**) Correction between ORGs signature, JAVELIN, Tumor inflammation, IMmotion 150 T-effector response and IMmotion 150 Myeloid gene expression signatures respectively. (**D**) Correlation between ORGs signature, immune checkpoint (ICI) genes (topper right) and tumor inflammation signature (TIS) genes (lower left) respectively. (**E**) Correlation between ORGs signature and pan-cancer T cell inflamed score. (**F**) Heatmap of effect genes of CD8+ T cell, dendritic cell (DC), macrophage, natural killer (NK) cell and type 1 T helper (Th1) cell between high-risk and low-risk ORGs signature cohorts. (**G**) Correlation between ORGs signature and the individual genes included in the T cell inflamed signature.

ORGs signature was found to be closely correlated with a large number of immunomodulators. We found that effector genes of Chemokine, Immunostimulator, MHC and Receptor were up-regulated in the high ORGs signature group (Figure 5A). In addition, TILs infiltration was generally positively correlated with ORGs signature by ssGSEA algorithm (Figure 5B). In addition, the four immune-related pathways including JAVELIN signature, Tumor inflammation signature (TIS), IMmotion150 T-effector (Teff) signature and IMmotion150 Myeloid signature were all significantly corrected with ORGs signature cohort (Figure 5C). These results could help immunotherapy efficacy predicting. What’s more, ORGs signature was positively correlated with 18 immune checkpoint inhibitors (ICIs) genes (Figure 5D, right upper) and 22 TIS genes (Figure 5D, left bottom). ORGs signature was also positively correlated with the pan-cancer T cell inflamed score (R=0.19, P=1.9e-0.5) (Figure 5E). Similarly, ORGs signature was positively correlated with the effector genes of these tumor-infiltrating immune cells (TIICs) (Figure 5F). Consistently, ORGs signature was found to be positively correlated with a majority of ICIs including LAG-3, CTL4, PDCD1, HAVCR2, PVR, CD80, CD86 and so on (Figure 5G).

### Analysis of immune status by GO and GSEA analysis

**Figure 6:**
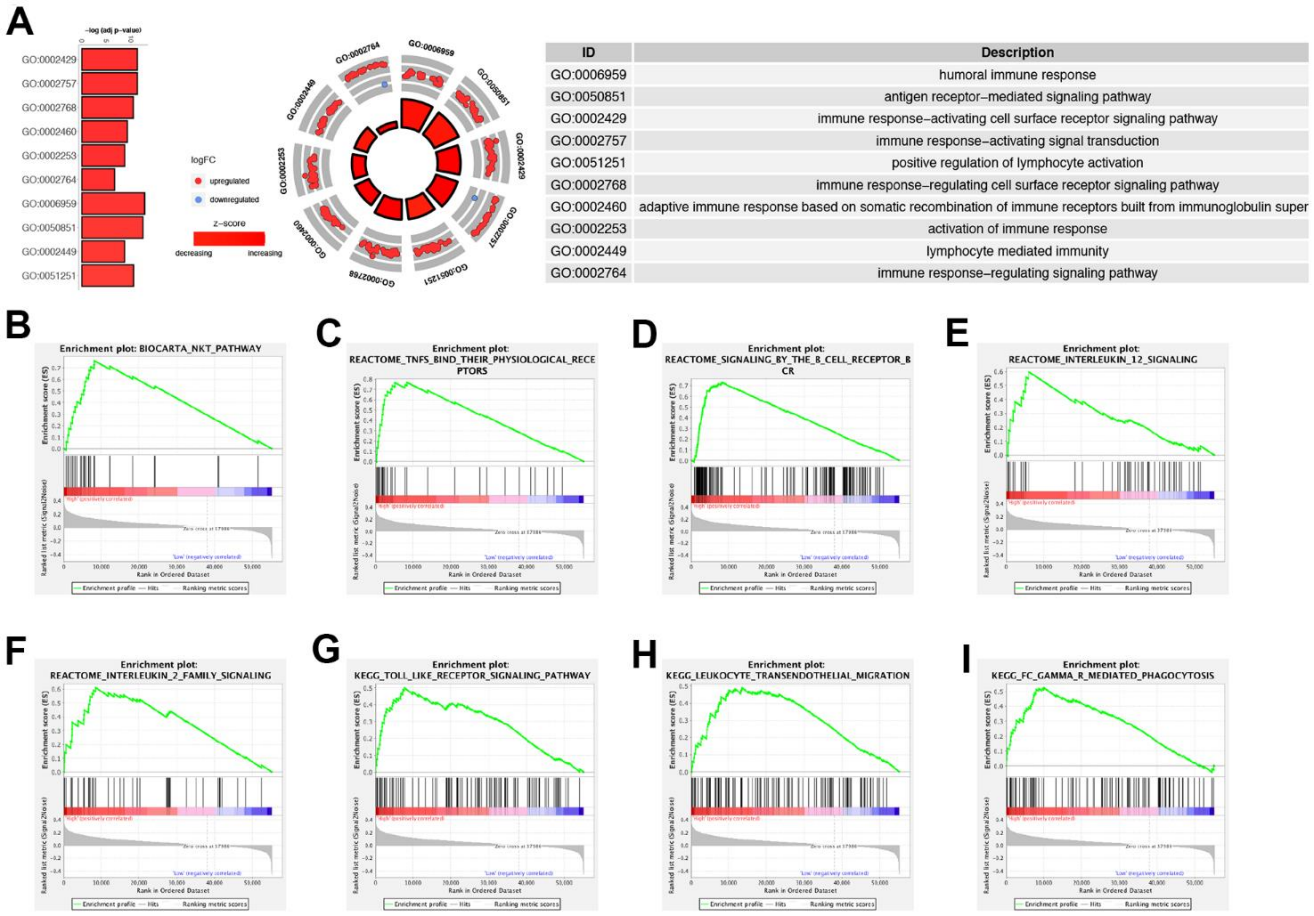
**Functional enrichment analysis and GSEA analysis.** (**A**) GO circle plots displayed scatter plots of log fold change (logFC) for the most statistically significant GO terms. Red dots represented up-regulated genes and blue dots represented down-regulated genes. The inner circles displayed z-scores calculated as the number of up-regulated genes minus the number of down-regulated genes divided by the square root of the count. (**B**–**I**) GSEA analysis exhibited that Biocarta NAK pathway (**B**), Reactome TNFs their physiological receptors (**C**), Reactome signaling by the B Cell receptor bcr (**D**), Reactome interleukin 12 signaling (**E**), Reactome interleukin 2 family signaling (**F**), KEGG toll like receptor signaling pathway (**G**), KEGG leukocyte trans-endothelial migration (**H**) and KEGG fc gamma r mediated phagocytosis (**I**) were enriched in the high-risk group compared with low-risk group.

Functional enrichment analysis showed that GO enrichment terms for the up-regulated and down-regulated ORGs signature by GOCircle plot (Figure 6A). Furthermore, we performed GSEA analysis and the results showed that Biocarta NAK pathway, Reactome TNFs their physiological receptors, Reactome signaling by the B Cell receptor bcr, Reactome interleukin 12 signaling, Reactome interleukin 2 family signaling, KEGG toll like receptor signaling pathway, KEGG leukocyte trans-endothelial migration and KEGG fc gamma r mediated phagocytosis were enriched in high-risk cohort compared with the low-risk cohort (Figure 6B–6I). In a word, the ORGs prognostic risk signature model was closely correlated with the immune status of PRAD patients.

### The role of ORGs signature on the single cell level

**Figure 7:**
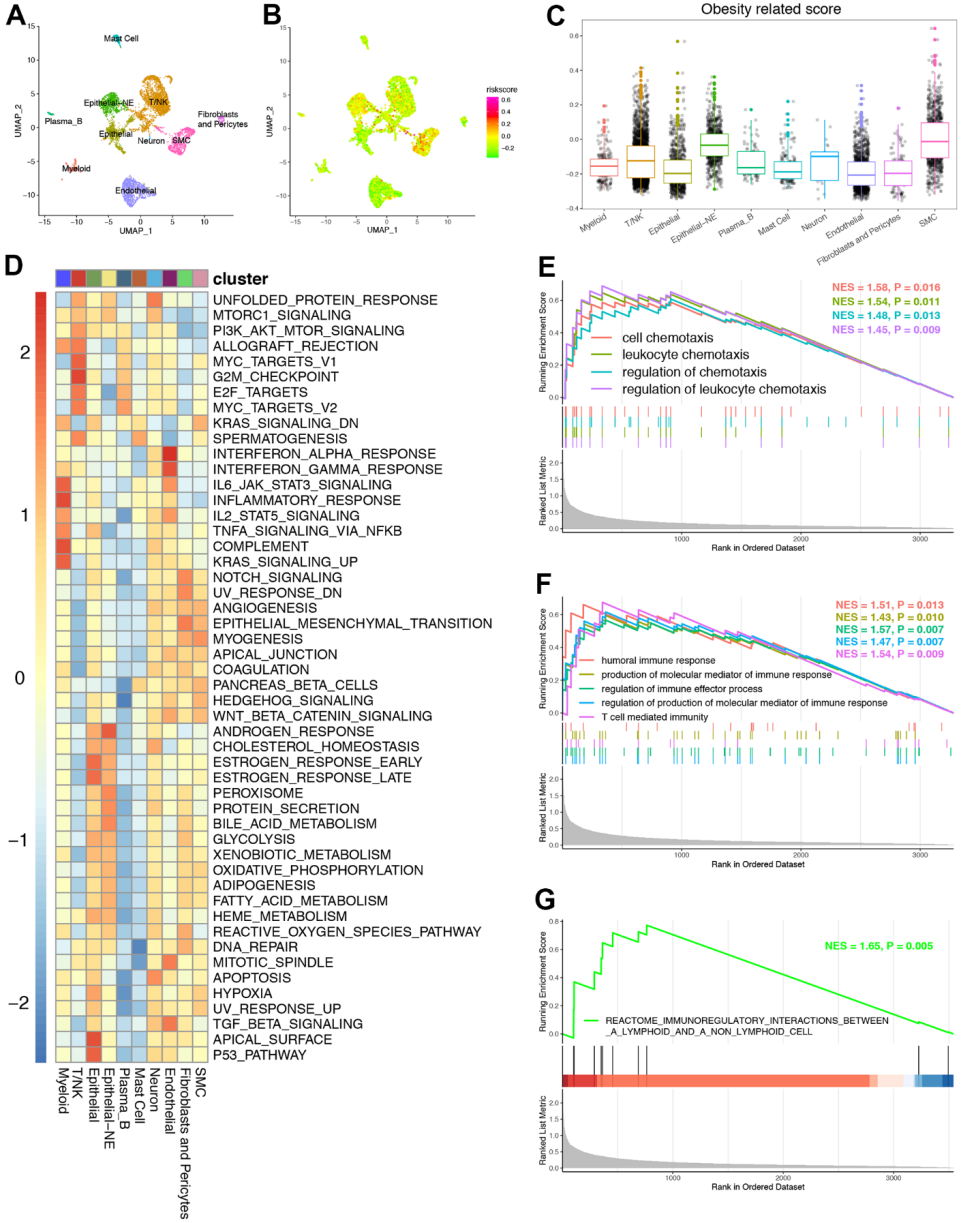
**The role of ORGs signature on the single cell level.** (**A**) UMAP plots of GSE157703 and each cluster were visualized and marked by different cell types. (**B**) Distribution of ORGs signature on the single cell level. (**C**) The expression of ORGs on the single cell level. (**D**) The heatmap revealed that significant connection probability of ORGs related signaling pathways on single cell level. (**E**) Gene ontology (GO) enrichment of chemokine related signatures identified by gene set enrichment analysis (GSEA) on the single cell level. (**F**) Gene ontology (GO) enrichment of T cell activation related signatures identified by gene set enrichment analysis (GSEA) on the single cell level. (**G**) Kyoto Encyclopedia of Genes and Genomes (KEGG) enrichment of T cell activation related signature identified by GSEA on the single cell level.

The above analysis was based on a large number of RNA-seq, so we further used scRNA-seq to determine whether our ORGs signature had immune predictive value on the single cell level. Figure 7A showed that the PRAD samples from GEO datasets were annotated into Mast cell, Epithelial-NE, T/NK, Plasma_B, Epithelial, Neuron, SMC, Fibroblasts and Pericytes, Myeloid and Endothelial. It was obvious that ORGs signature was specifically high in Epithelial-Nk, T/Nk and SMC cells (Figure 7B, 7C). Meanwhile, Figure 7D revealed that significant connection probability of ORGs related signaling pathways in cell clusters which showed specifically high ORGs signature. Furthermore, we chose epithelial cell for analysis. Chemotaxis related pathways including cell chemotaxis, leukocyte chemotaxis, regulation of chemotaxis and regulation of leukocyte chemotaxis were significantly up-regulated in the high ORGs signature group (Figure 7E). What’s more, immune related pathways were all up-regulated in the high ORGs signature group both in GO (Figure 7F) and KEGG (Figure 7G) analysis.

### The expression levels of ORGs in PRAD cell lines detected by RT-qPCR

**Figure 8:**
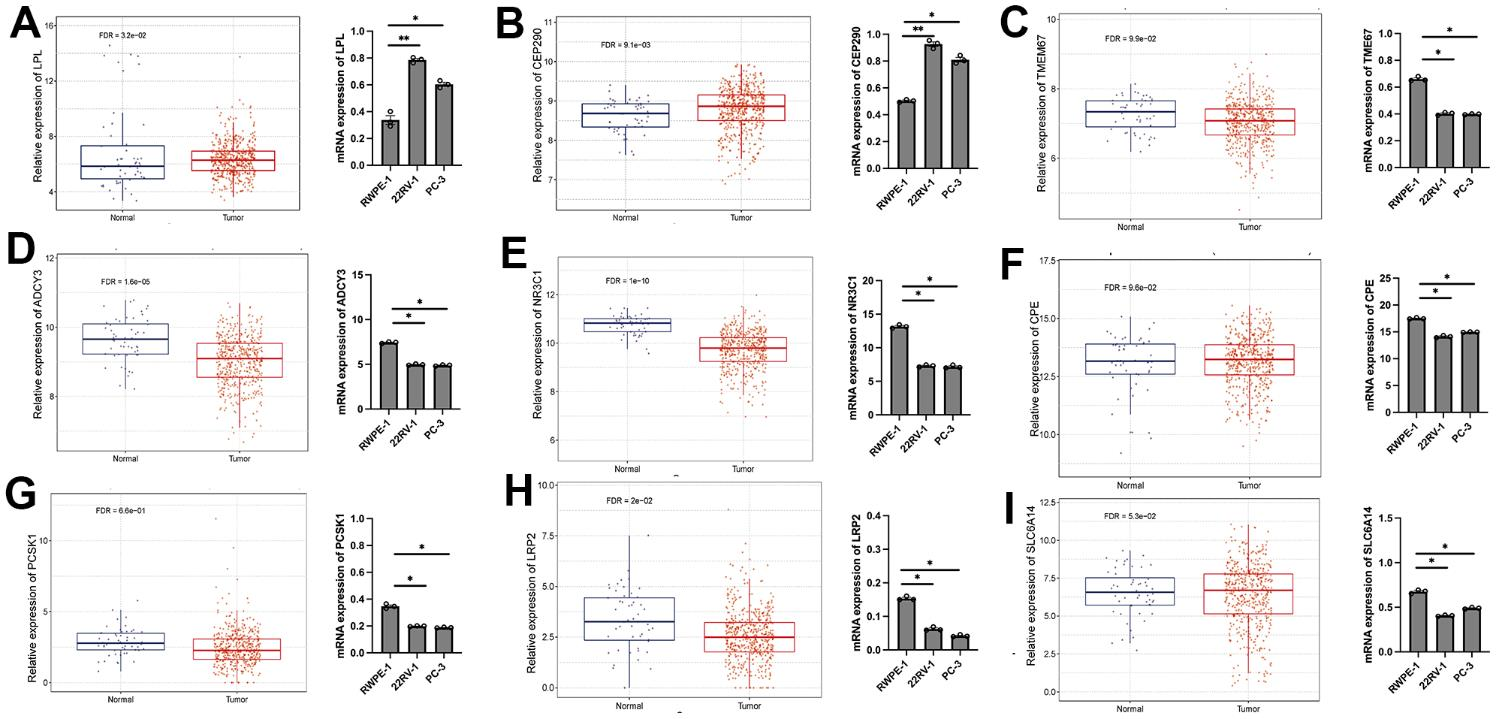
The expression levels of LPL (**A**), CEP290 (**B**), TMEM67 (**C**), ADCY3 (**D**), NR3C1 (**E**), CPE (**F**), PCSK1 (**G**), LRP2 (**H**) and SLC6A14 (**I**) in LUAD cell lines and normal prostate cell lines detected by RT-qPCR. Human prostate cell line: RWPE-1. PRAD cell line: 22RV1 and PC-3. *p < 0.05; **p < 0.01; ***p < 0.001; ****p < 0.0001. ns, not significant.

We performed cytological verification on several ORGs (LPL, CEP290, TMEM67, ADCY3, NR3C1, CPE, PCSK1, LRP2 and SLC6A14) in the ORGs signature. LPL’s higher expression in PRAD cell than normal prostate cell confirmed by our RT-qPCR, which was consistent with TCGA analysis result (Figure 8A). Similarly, several other ORGs were also verified to be consistent with TCGA analysis results by RT-qPCRs (Figure 8B–8I).

## DISCUSSION

In recent years, more and more evidence show that obesity is a related risk factor for a variety of malignant tumors, including invasive PCa [2, 15]. The proteins encoded by LEP and ANGPT1 may have functions other than adipose tissue itself. Leptin receptor is expressed in PRAD and leptin staining is significantly increased [37]. Angiopoietin 1 and its receptor Tie-2 are also found in PRAD cells and their capillaries, which could induce tumor angiogenesis [38, 39]. But all these studies only focused on one or a small number of new genes, and the comprehensive relationship between ORGs, cancer immunity and immunotherapy was lacking.

At present, many studies have shown the complexity and co-regulation characteristics of TME by analyzing the relationship between TME and gene list. For example, Li et al. generated m6A modified clusters based on 24 m6A modified genes, and associated them with TME and immunotherapeutic efficacy of renal cell carcinoma [40]. The other study divided bladder cancer patients into 5 hypoxia response modes and generated individual hypoxia response modes. Li et al. also found that cuproptosis patterns depicted different TME phenotypes in bladder cancer [30]. Wan et al. systematically analyzed their relationship with glioma prognosis and immunotherapy efficacy by constructing two ferroptosis groups [41]. To our knowledge, this is the first study to systematically link ORGs with TME, prognosis and immunotherapy efficacy in PRAD. We identified high- and low-risk clusters of ORGs, and found that there were different survival outcomes behind the two clusters. In addition, high-risk cluster and low-risk cluster represent different TME phenotypes and immune response rates. For individual PRAD patients, we also constructed ORGs risk score and ORGs signature for prognosis and cancer immune prediction respectively.

PRAD is one of the most common solid tumors in men worldwide [42]. With the increase of incidence rate of PRAD, it poses a huge threat to human health and economy. Prostate specific antigen (PSA) testing is widely used in the United States and Europe, but due to the high rate of false positives, misdiagnosis and overtreatment, it soon became obsolete. The significant increase and decrease in PRAD mortality can be attributed to these factors [43, 44]. In addition, studies have found that metastatic castration-resistant prostate cancer (mCRPC) patients with high tumor mutational burden (TMB) have better overall survival (OS) after receiving ICIs treatment [45]. Graf et al. evaluated a cohort of patients treated with ICIs or taxanes through a comparative effectiveness study, and found that the time to next therapy and OS of patients with high TMB treated with ICIs were improved compared with those treated with taxanes [45]. In addition, the largest single study evaluated MSI-H or mismatch repair defect mCRPC patients receiving anti-PD-1 or anti-PD-L1 treatment, and found that PSA levels in 6 of 11 patients decreased by 50% or more [46]. A trend for superior PFS and OS was observed in this cohort of PRAD patients with bone-predominant disease compared with those assessable for both response evaluation criteria in solid tumors and PD-L1 [47]. These clinical trials have promoted the approval of ICIs for PRAD and revealed the important role of ICIs. But, at present, not all patients responded to the treatment of ICIs in the trial, which indicated the urgent need to find biomarkers to predict the efficacy of ICIs.

Recent studies have revealed that TME promotes the development of cancer biology and immunology by affecting the immune system of the host [48–51]. Patients with immunosuppression micro-environment and less TILs had poor response rate to ICIs treatment and poor OS [45]. In addition, the study believed that distinguishing non-inflammatory tumors from inflammatory tumors can not only predict the efficacy of ICIs, but also transform “cold” into “hot”, so as to obtain higher efficacy of ICIs [48]. Many studies on PRAD have linked the signatures of pyroptosis and ferroptosis with TME and immunotherapy efficacy [52–54]. In this study, we first generated a ORGs signature of accurate TME phenotype prediction. Importantly, this result was also well verified in the external data set, which made our results more reliable. More importantly, our ORGs signature model could directly predict the efficacy of immunotherapy, which was crucial for accurate ICIs treatment of PRAD.

This study had some limitations. First of all, all our results were from a retrospective public database, which needs to be further verified by prospective research. In addition, although we have verified the ORGs signature in TME and immunotherapy through external data, the specific therapeutic mechanism needs to be further studied *in vivo* and *in vitro*.

## CONCLUSIONS

ORGs patterns depict different TME phenotypes in PRAD. ORGs risk score and signature have potential role for predicting prognosis and the efficacy of immunotherapy that could guide accurate medicine.

## Supplementary Material

Supplementary Figure 1

Supplementary Tables
