# Supplementary material for: Identification and validation of obesity related genes signature based on microenvironment phenotypes in prostate adenocarcinoma
Source: Aging (Albany NY). 2023 Oct 2;15(19):10168–92. doi: 10.18632/aging.205065 (PMC10599753; doi:10.18632/aging.205065)
Supplement: Supplementary Figure 1 [file aging-15-205065-s001.pdf]

SUPPLEMENTARY FIGURE

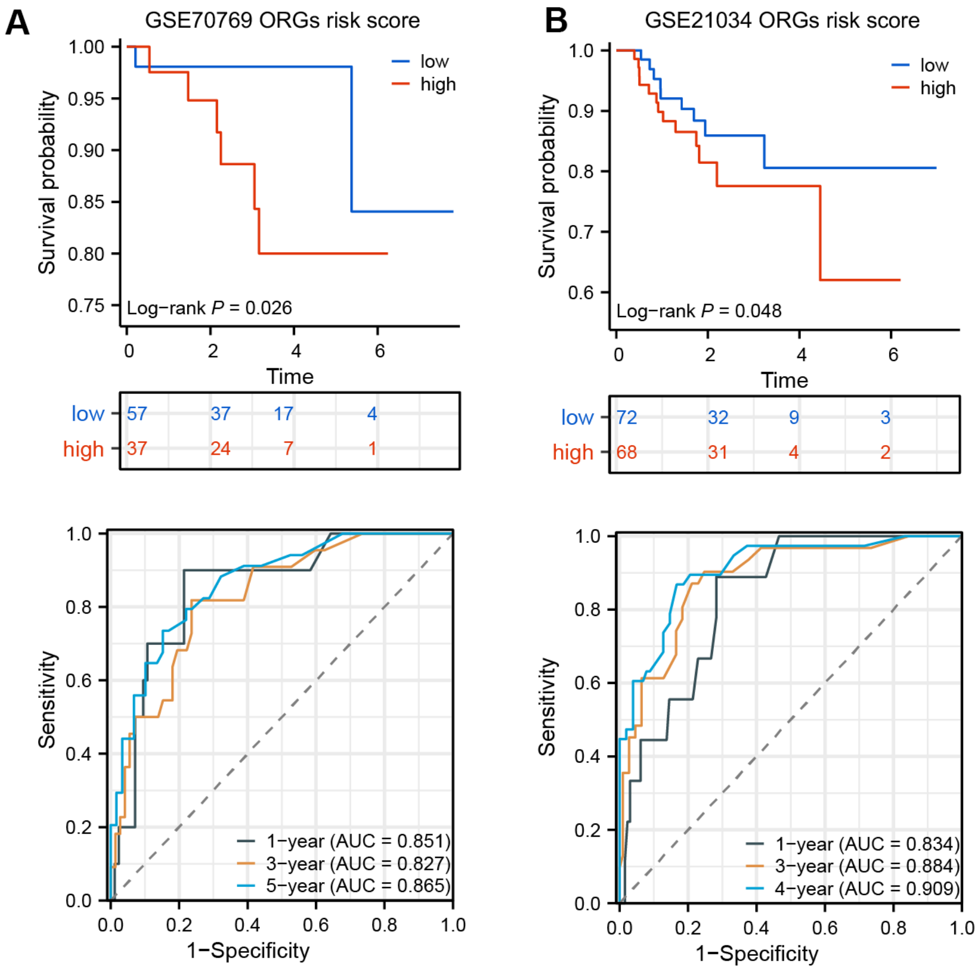

Supplementary Figure 1. Validation of the accuracy of the ORGs risk score algorithm for predicting BCR probability of GSE70769 and GSE21034 datasets. (**A**, **B**) Kaplan-Meier curve analysis was used to analyze the BCR of patients with high and low ORGs risk score. Time-dependent ROC curves were used to compare the 1-, 3-, 5-year BCR between high and low ORGs risk score.
