# Supplementary material for: Identification and validation of obesity related genes signature based on microenvironment phenotypes in prostate adenocarcinoma
Source: Aging (Albany NY). 2023 Oct 2;15(19):10168–92. doi: 10.18632/aging.205065 (PMC10599753; doi:10.18632/aging.205065)
Supplement: Supplementary Tables [file aging-15-205065-s002.pdf]

SUPPLEMENTARY TABLES

Supplementary Table 1. ORGs were summarized in this study.

LEP
LEPR
MC4R
POMC
PCSK1
ADCY3
SIM1
BDNF
NEGR
TCF7L2
IRS1
FTO
RPTOR
MAP2K5
NPY
SLC6A4
MCHR1
ADIPOQ
LPL
PPARG
IGF2/H19
HSD2
GR
SREBF1
ABCA1
TNFA
CD36
PHGDH
TOMM20
ABCG1
PHOSPHO1
PER3
HIF3A
TXNIP
PEG3
LY86
ADRB3

Supplementary Table 2. The primers for q-PCR in this study.

	Primer	Sequence (5' to 3')
1	LPL For	TCATTCCCGGAGTAGCAGAGT
2	LPL REV	GGCCACAAGTTTTGGCACC
3	CEP290 For	AGATGCTCACCGAACAAGTAGA
4	CEP290 REV	ATGAGTCTGTTGAGAAAGGGTTG
5	TMEM67 For	CTTGGCTGTTTTATGGAGACCA
6	TMEM67 REV	ACCTCCTTCTAAAGTTTGCCAC
7	ADCY3 For	TTCTCCGAGCCCGAATACTC
8	ADCY3 REV	GACTCCGGCACGAAAGTCA
9	NR3C1 For	ACAGCATCCCTTTCTCAACAG
10	NR3C1 REV	AGATCCTTGGCACCTATTCCAAT
11	CPE For	CATCTCCTTCGAGTACCACCG
12	CPE REV	CCGTGTAAATCCTGCTGATGG
13	PCSK1 For	CTGGATGGCATTGTGACGGAT
14	PCSK1 REV	GCCCCAGCTTGCACTGTAAA
15	LRP2 For	GTTCAGATGACGCGGATGAAA
16	LRP2 REV	TCACAGTCTTGATCTTGGTCACA
17	SLC6A14 For	ACCGTGGTAACTGGTCCAAAA
18	SLC6A14 REV	CGCCTCCACCATTGCTGTAG
